# Supplementary material for: Hecw controls oogenesis and neuronal homeostasis by promoting the liquid state of ribonucleoprotein particles
Source: Nat Commun. 2021 Sep 16;12:5488. doi: 10.1038/s41467-021-25809-8 (PMC8446043; doi:10.1038/s41467-021-25809-8)
Supplement: Supplementary file 1 — Supplementary Information [file 41467_2021_25809_MOESM1_ESM.pdf]

Supplementary Information

Hecw controls oogenesis and neuronal homeostasis by promoting the liquid state of ribonucleoprotein particles

Fajner et al.

Supplementary Figures 1-8

Supplementary Tables 1-4

Supplementary Figures

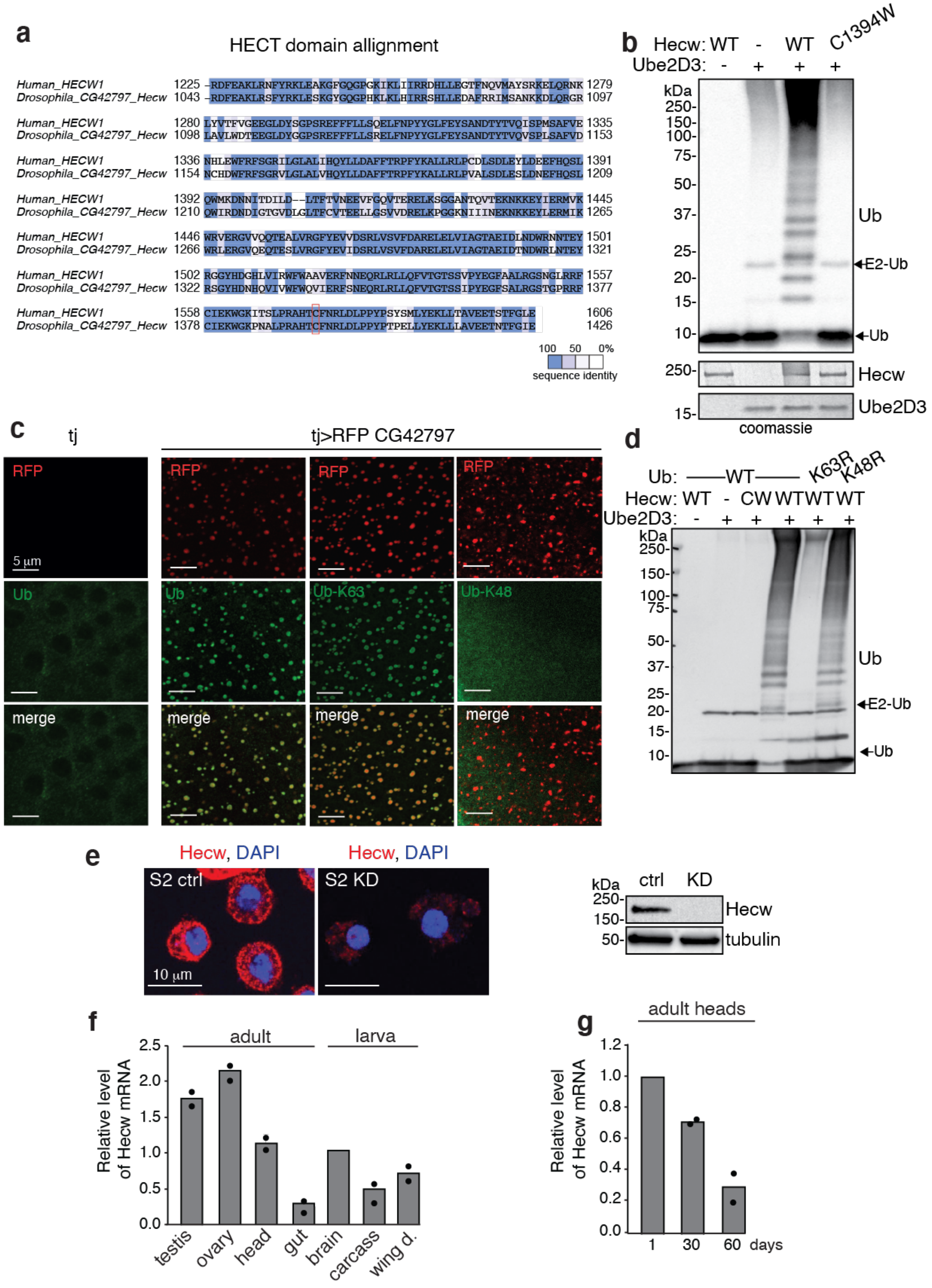

Supplementary Figure 1: Hecw is a K63-specific ubiquitin E3 ligase.

(**a**) Amino acid sequence alignment of the catalytic HECT domain of human HECW1 and *Drosophila* CG42797, color-coded according to sequence conservation. The catalytic cysteine is highlighted in the red box. ClustalW was used to create the sequence alignment of HECT between human and *Drosophila* proteins. (**b**) *In vitro* self-ubiquitination assay with the indicated recombinant proteins, analysed by IB with anti-Ub antibody. The reaction was quenched after 60 minutes. C1394W, a catalytic inactive mutant harbouring the same mutation present in Hecw^{CI}. Coomassie shows equal loading (lower panel) (n=3). (**c**) IF analysis of *Drosophila* follicle cells with the indicated fluorescent tag and antibodies. RFP-CG42797 is overexpressed in follicle cells with the *traffic jam-GAL4* driver (tj). RFP-Hecw localises in puncta that co-localises with a pan ubiquitin antibody (FK2, Ub) and an antibody that specifically recognises K63-linked chains, but not with an anti-K48 antibody. Left panels, control (tj driver only) shows no RFP signal and no accumulation of ubiquitinated puncta. Confocal images are shown. (n=2). Scale bar: 5 µm. (**d**) *In vitro* self-ubiquitination assay with the indicated recombinant proteins and ubiquitin wild-type, K63R or K48R mutants. The experiment was repeated three times with identical results. (**e**) IF analysis of *Drosophila* S2 cells untreated (ctrl) or depleted of Hecw by treatment with dsRNA (KD) for 48 hours. Hecw, red; DAPI, blue. Scale bar, 10 µm. Right panel, lysates from the same cells were IB as indicated. (**f**) *Hecw* mRNA expression measured in the indicated *Drosophila* tissues by qPCR. Expression levels relative to larval brain are expressed as mean calculated over two experiments with three technical replicates. Dots represents the mean of the single replicate. (**g**) *Hecw* expression measured by qPCR in adult heads of the indicated age. Expression levels relative to 1-day-old flies are expressed as mean calculated over two experiments with three technical replicates. Dots represents the mean of the single replicate.

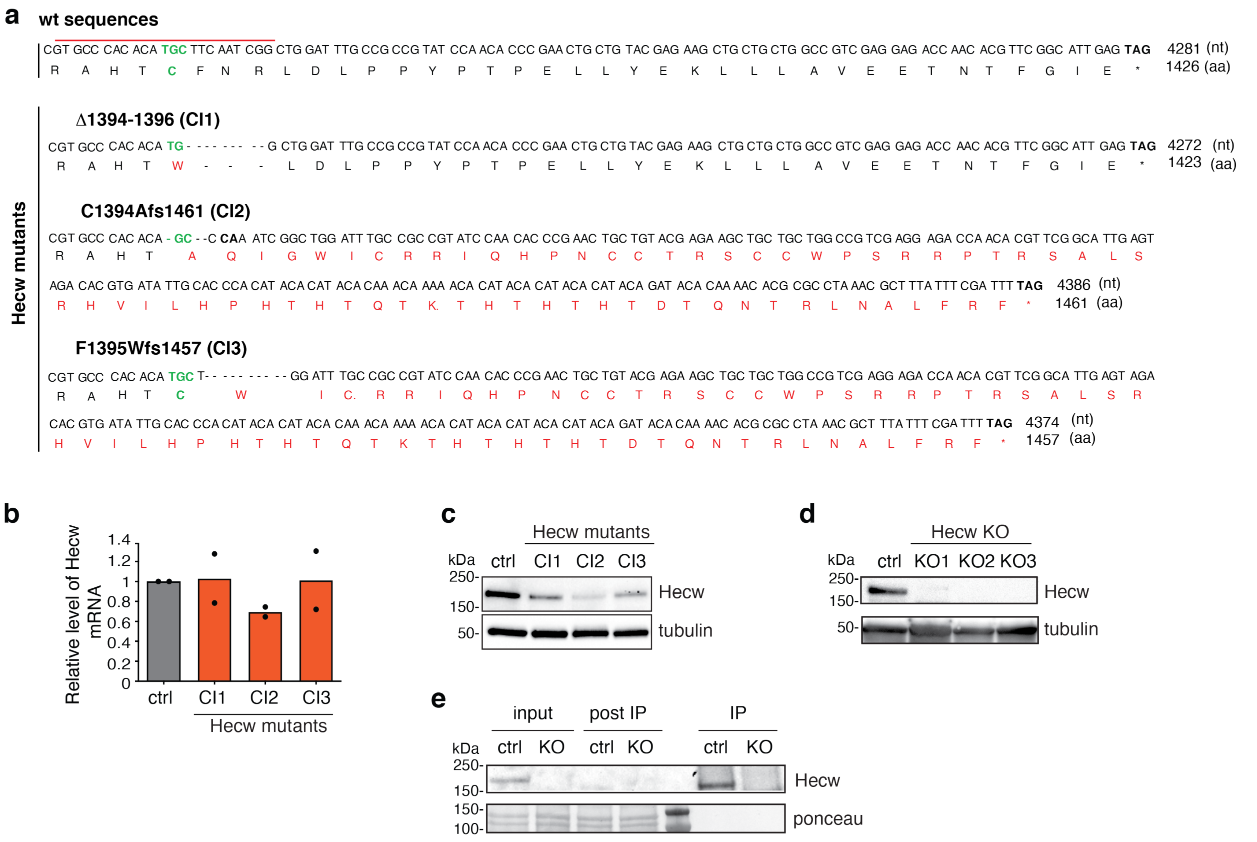

Supplementary Figure 2: Characterisation of Hecw catalytic mutants generated by CRISPR/Cas9 technique.

(**a**) Nucleotide and amino acid sequences of the mutants generated. Top panel: wild-type (wt) sequence of nucleotides (nt) and amino acids (aa). The catalytic cysteine in green and the stop codon is indicated with an asterisk. The red line indicates the region targeted by the Cas9 guide. In mutant sequences, deletions are indicated with dashes and amino acids that differ from the wild-type are in red. (**b**) mRNA levels of *Hecw* were measured by qPCR in larval brains of the mutated lines. Expression levels relative to wild-type larval brain are expressed as mean calculated over two experiments with three technical replicates. Dots represents the mean of the single replicate. (**c**) IB analysis of larval brains of the mutated lines. The expression levels of Hecw mutant proteins are reduced in comparison with wild-type control (yw). (**d**) IB analysis of adult ovaries of the Hecw^{KO} lines. (**e**) 0.5 mg of ovary lysates from the indicated lines were IP with the anti-Hecw antibody and IB as indicated. Post IP, supernatant post IP.

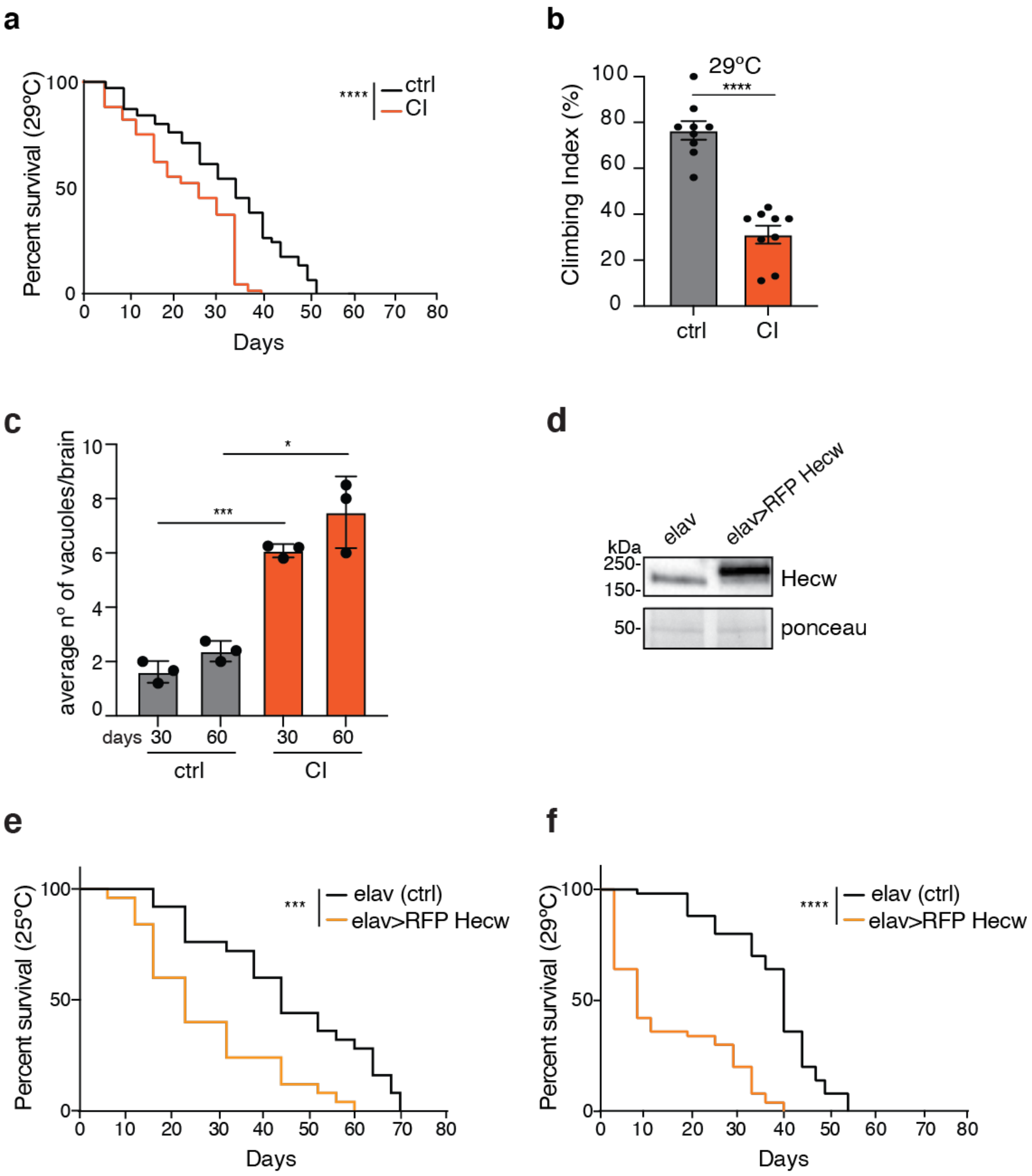

Supplementary Figure 3: Hecw depletion and overexpression cause reduced longevity and premature motor function impairment.

(**a**) Survival curve of the indicated genotypes. Percentage of survival was calculated over 100 animals at 29°C. *Hecw*^{CI} (CI) shows a significant decrease in lifespan compared with the control line (****P<0,0001 with log-rank test). (**b**) Negative geotaxis assay performed on 12 day-old flies at 29°C. Results are expressed as climbing index mean ± s.e.m (*n*=15 animals/9 independent groups). Mutant flies show a climbing deficit, ****P<0,0001 with two-tailed Mann

Whitney test. (**c**) Quantification of vacuoles with diameter >2 µm in 30-day-old fly brains of the indicated genotypes. Results are expressed as mean ± s.e.m of three biological replicates. (n=5 animals/genotype/replicate) **P<0,01 with two-tailed *t*-test. (**d**) IB analysis of adult head lysates with the indicated antibodies. RFP-Hecw induced by the *elav-GAL4* driver (elav> RFP Hecw) runs at higher mw in comparison with wild-type Hecw protein expressed in the control line (elav driver only). (**e, f**) Survival curve of the indicated genotype at 25°C (**e**) and 29°C (**f**). Percentage of survival was calculated over 50 animals per genotypes. Flies with overexpressed Hecw in neurons show a significant decrease in their lifespan compared with the control line, *** P=0,0003 (**e**), ****P<0,0001 (**f**) with log-rank test.

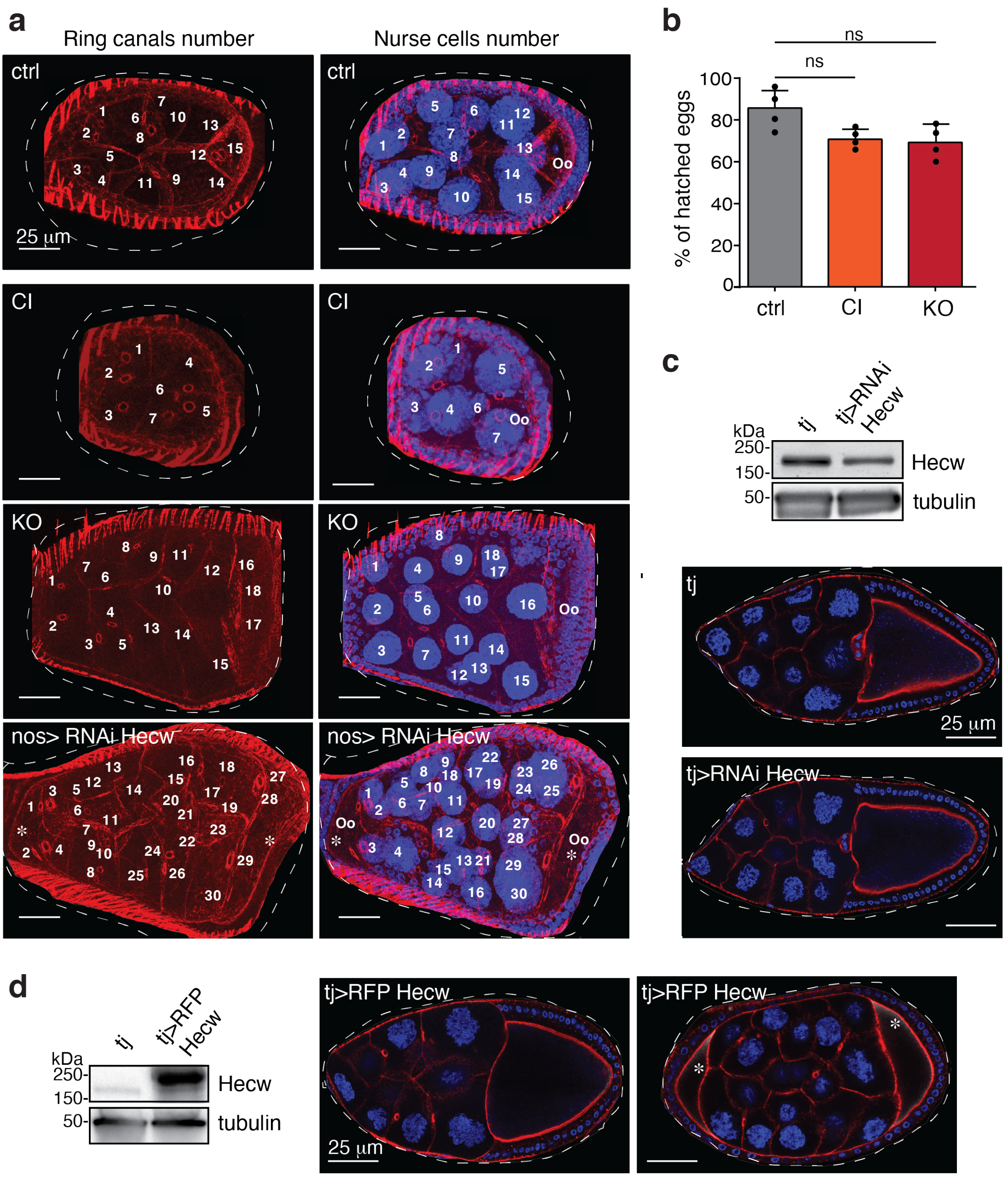

Supplementary Figure 4: Defective egg chambers upon Hecw perturbation.

(**a**) Confocal analysis of ovaries of the indicated genotypes. Maximal projection of the z-stack images is shown. Total number of nurse cells and ring canals were counted to assess the amount of germline cells and cell division. Observed defects include a reduced number of nurse cells (CI, *Hecw*^{CI} mutant is shown as an example), an increased number of nurse cells (KO, *Hecw*^{KO} is shown as an example), and compound egg chambers (Hecw germline-specific knock down *nos>RNAi Hecw* is shown as an example; white asterisks indicate the two oocytes). Phallodin, red; DAPI, blue. (n=2). Scale bar: 25 µm. (**b**) Hatching rate of eggs laid in 24 hours by 20-day-

old flies of the indicated genotypes. Results are expressed as mean of number of laid eggs ± SD calculated over four experiments (*n*=6 females/exp), ns by Mann Whitney test. (**c**) IB analysis of ovary lysates from control (follicle cells driver only, *traffic jam-GAL4* tj) and Hecw knock down (*tj>UAS-RNAi Hecw*) 3-day-old flies. Note that depletion appears limited as follicle cells represents 20-25% of the single egg chamber. Bottom, IF analysis of the same egg chambers. Red, phalloidin; blue, DAPI. Scale bar: 25 μm. Ovaries with Hecw depletion only in follicle cells do not present oogenesis defects. (**d**) IB and IF analysis of the indicated genotype, as in c. Most of the egg chambers with ectopic Hecw expression in follicle cells (tj>UAS RFP Hecw) do not present oogenesis defects (central panel) except for 3,5% of them that present compound egg chambers (right panel; white asterisks indicate the two oocytes). See also Supplementary table 1 for the quantification.

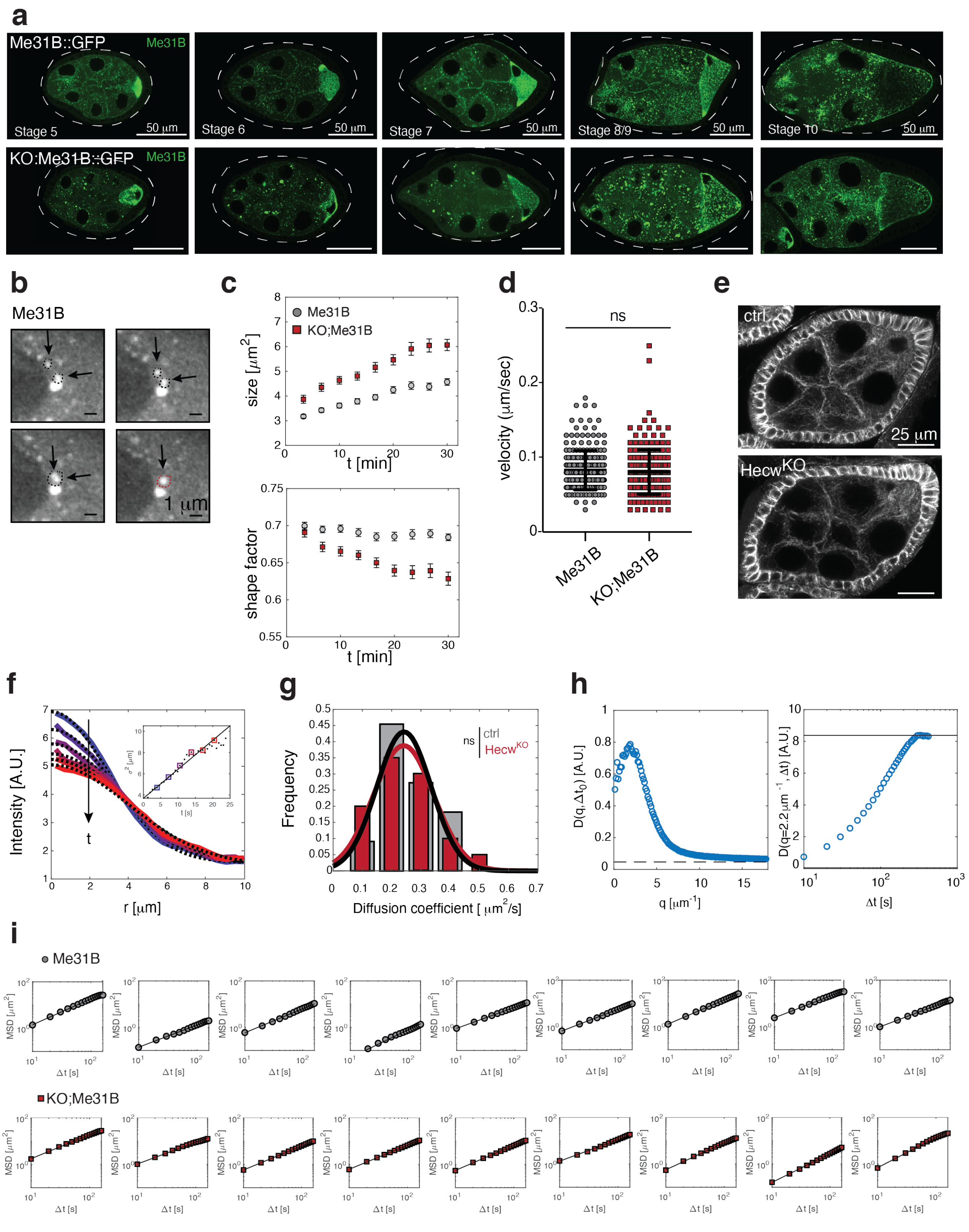

Supplementary Figure 5: Analysis of RNPs mobility and cytoplasm diffusion.

(**a**) Examples of egg chambers from 3-day-old flies at different stages of development. Egg chambers of *Hecw^KO; Me31B::GFP* animals show enlarged RNPs. Scale bar: 50 μm. IF experiment was repeated three times with identical results. (**b**) Fusion of RNPs (indicated by arrow heads

and dashed circles in sequential frames) in *Me31B::GFP* egg chambers. Scale bar 1 μm. (**c**) Coarsening of RNPs during *ex-vivo* observation (n=3). Temporal evolution of size (upper panel) and shape factor $4\pi A/P^2$ (bottom panel) of the RNPs. A progressive increase in the average size of the RNPs (coarsening) is observed both in wild-type (grey dot, *Me31B::GFP*) and $Hecw^{KO}$ (red squares, $Hecw^{KO}$; *Me31B::GFP*) flies ovaries. In wild-type egg chambers, the coarsening process takes place without any significant change in the geometry of the granules, which grow while maintaining their approximately spherical shape. On the contrary, in $Hecw^{KO}$ egg chambers, the increase of the average size of the granules is accompanied by a marked decrease of the shape factor, indicating the formation of irregularly shaped aggregates. (**d**) Quantification of the speed of tracked single RNP by live imaging analysis. The velocity of control (Me31B::GFP) and $Hecw^{KO}$ (KO;Me31B) particles is not statistically different (v = mean±SD, v_{wt} = 0,0829 ± 0,026 μm/s, v_{ko} = 0,0804 ± 0,0298 μm/s. P=0,3302, ns by two- tailed Mann Whitney test). Statistical analysis was performed over 3 biological replicates for a total of n=220 particles for the control and n=213 for $Hecw^{KO}$ (11 egg chambers/sample). (**e**) Microtubule-dependent movement of Me31B is not affected by Hecw. IF analysis to detect the microtubule marker α-tubulin in control (*Me31B::GFP*) and $Hecw^{KO}$ ($Hecw^{KO1}$;*Me31B::GFP*) egg chambers dissected from 3-day-old flies. (n=2). Scale bar: 25 μm. (**f,g**) FRAP analysis performed in the region of cytoplasm that does not include any RNP. (**f**) Continuous lines: azimuthally averaged intensity profiles measured at different times after photobleaching; dotted lines: best fitting Gaussian curves, enabling the estimation of a time-dependent width $\sigma(t)$. In the inset, $\sigma^2(t)$ is plotted *vs* time. A linear fit to the data (continuous line) enables estimating the diffusion coefficient D. (**g**) Bars: frequency distributions of the obtained diffusion coefficients, showing no statistically significant differences between $Hecw^{KO}$ and wild-type egg chambers. (P=0.9762, ns by two-tailed t-test). Continuous lines are Gaussian fits to the data. (**h**) Left panel: representative image structure function $D(q, \Delta t)$ obtained from DDM analysis as a function of the wavevector q for fixed $\Delta t = \Delta t_0 = 10\ s$. For large q the function attains a plateau value (dashed horizontal line) corresponding to the noise contribution B in Eq. (1). Right panel: for each value of q, the amplitude term $A(q)$ is estimated from the large Δt limit of $D(q, \Delta t)$ (continuous horizontal line). (**i**) Average mean square displacement $MSD(\Delta t)$ of RNPs determined with Differential Dynamic Microscopy (DDM). Each panel corresponds to a different egg chamber. 9 egg chambers/genotypes were analysed.

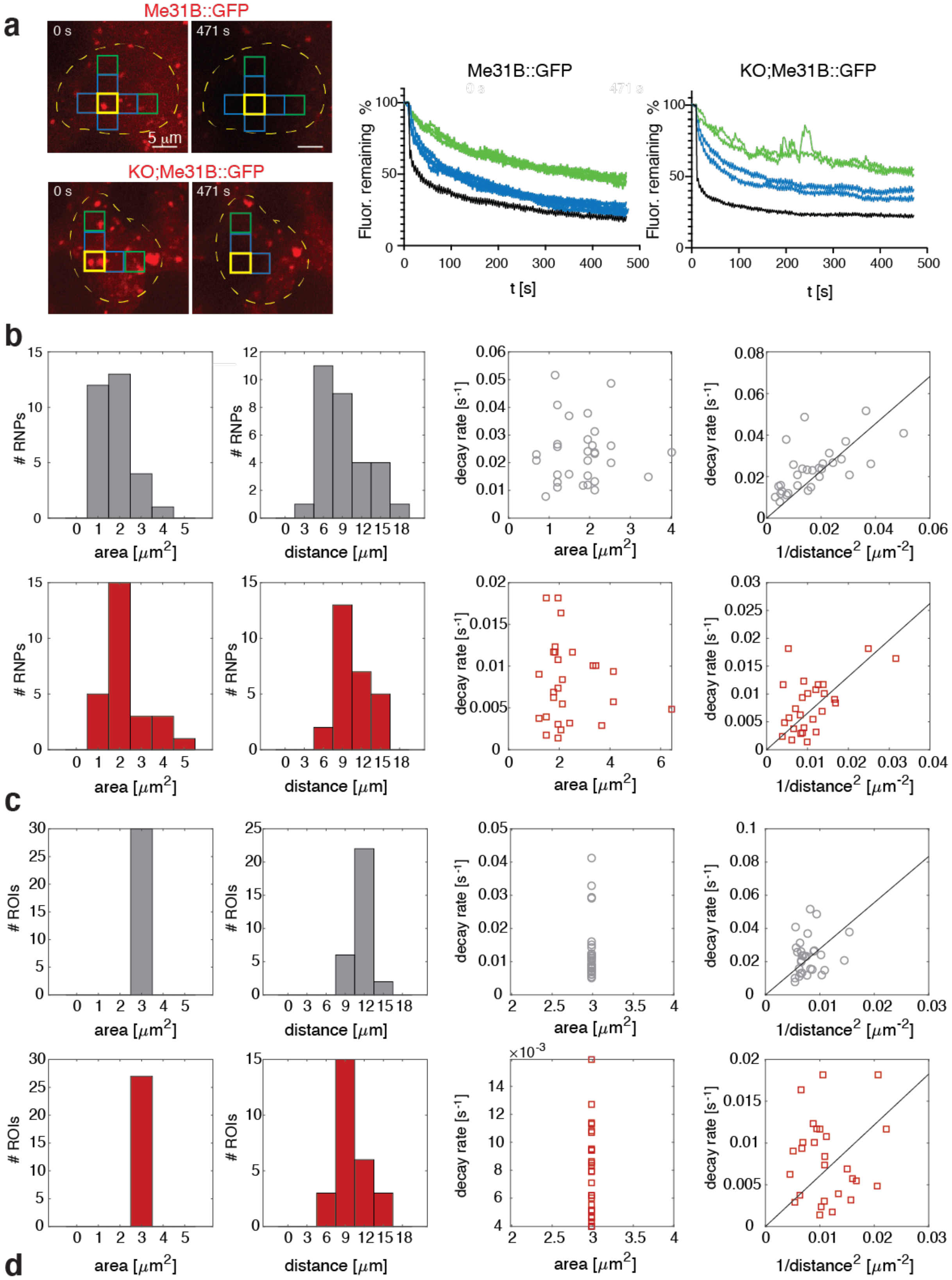

d

	Decay rate (WT)	Decay rate (KO)
1/distance²	ρ=0.5, p<10^3	ρ=0.75, p<10^5
Area	ρ=0.05, p=0.80	ρ=−0.01, p=0.96

Supplementary Figure 6: Free diffusion is not affected by the absence of Hecw.

(**a**) FLIP analysis performed in *Me31B::GFP* or *Hecw*^{KO} *Me31B::GFP* (KO;Me31B::GFP) fly ovaries. Yellow boxes indicate the regions continuously bleached for 7 minutes, blue and green boxes the neighbouring regions in which Me31B::GFP fluorescence is recorded every 2 seconds. Right panel: example of fluorescence fluctuation in neighbouring color-coded regions, plotted as percent relative to time 0. The black line corresponds to the bleached ROI. *n*=11 egg chambers/genotype. (**b, c**) Analysis of the size and spatial distribution of RNPs (first and second rows, **b**) and cytoplasm ROIs (third and fourth rows, **c**) considered in the FLIP experiment. From left to right in each row: frequency distribution of the areas, frequency distribution of the distances from the centre of the photobleached area, estimated decay rate *vs* area, estimated decay rate *vs* inverse squared distance. Grey bars and symbols refer to control, red bars and symbols refer to *Hecw*^{KO} cells. While in *Hecw*^{KO} egg chambers, RNPs are, on average, larger than those in wild-type egg chambers, no statistically significant correlation is observed between size and decay time within each group of nurse cells. Within each group of cells, there is a significant (negative) correlation between the distance from the bleached area and decay time. The distributions of distances in the two groups are not significantly different (second column of panel **b**). (**d**) Table reporting the Pearson correlation coefficient between fluorescence decay rate of the RNPs and inverse of the squared distance from the center of the photobleached area (first row) and between decay rate and area (second row). The corresponding p-values (obtained by two-tailed t-test) are also reported.

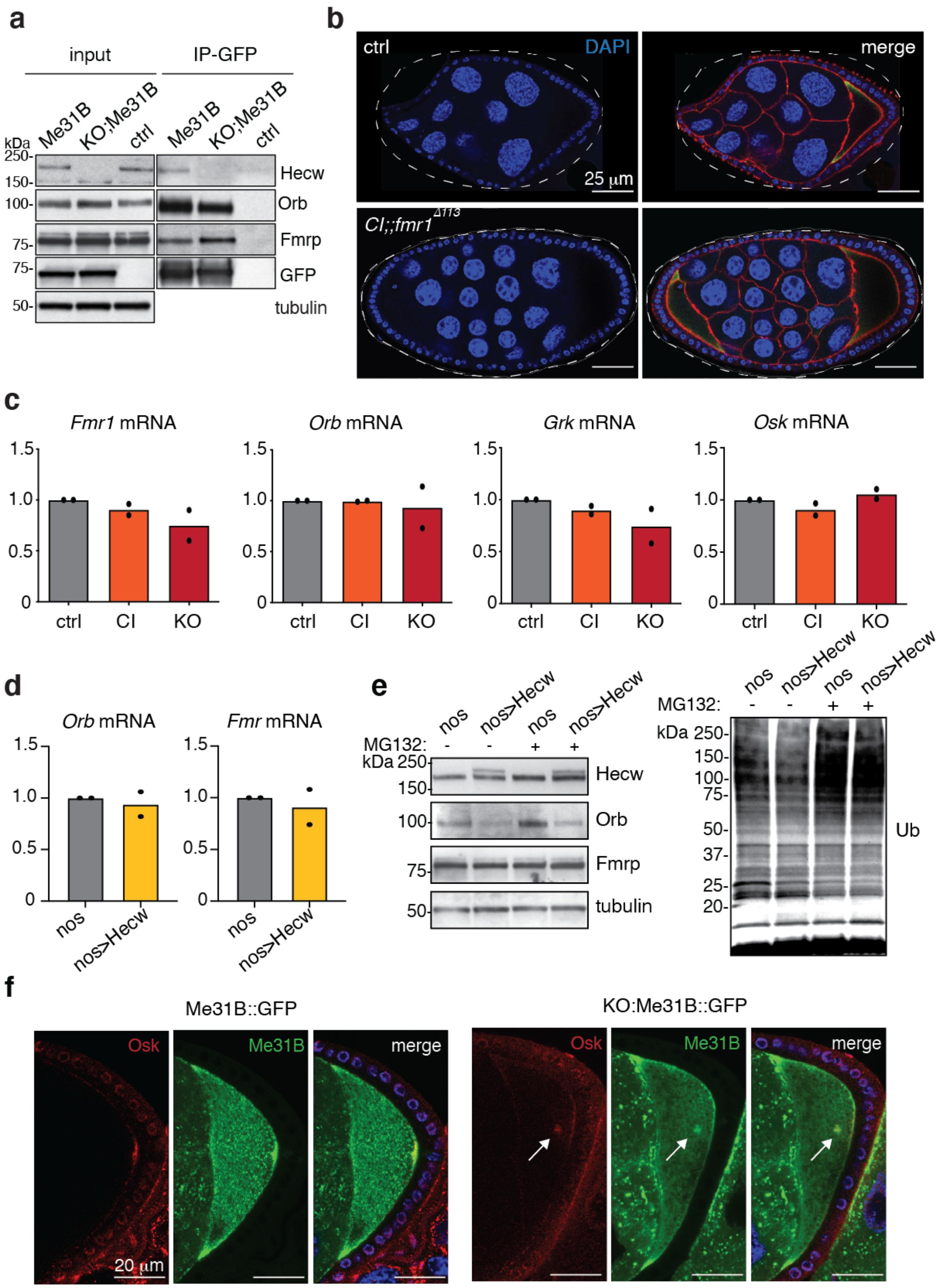

Supplementary Figure 7: Hecw interacts with RNP components.

(**a**) 0.5 mg of ovary lysates from the indicated lines were IP and IB as indicated. (**b**) IF analysis of egg chambers of 3-day-old control (yw) and *Hecw*^{CI} *;;Fmr1*$^{\Delta 113}$ double mutant (CI;;Fmr1)

animals. Double mutant shows no worsening of the single mutant phenotypes. Compound egg chamber is reported in the figure as an example. (n=3). Scale bar: 25 µm. (**c**) mRNA levels of *Fmr1, orb*, *grk* and *osk* was measured by qPCR in the adult ovaries of the indicated genotypes. The reported expression levels relative to control are expressed as mean calculated over two experiments with three technical replicates. Dots represents the mean of the single replicate. (**d**) mRNA levels of *Fmr1* and *orb* in the adult ovaries of the indicated genotypes, measured by qPCR as in c. (**e**) IB of control (driver only, nos) and RFP-Hecw overexpressing fly ovaries (nos>RFP Hecw) treated with 50 µM MG132 for 2 hours, and probed with the indicated antibodies. Left panel: Orb level decreases upon Hecw overexpression in the germline and does not increase upon proteasome inhibition. Right panel: IB with anti-Ub antibody shows Ub accumulation upon MG132 treatment. (**f**) IF analysis of 3-day-old wild-type and $Hecw^{KO}$ egg chambers. Green, Me31B::GFP marks RNPs. Upper panels: red, Osk localises at the posterior margin of the oocyte in wild-type control. Bottom panels: white arrows indicate mislocalised Osk in Hecw mutant flies. Blue, DAPI. (n=2). Scale bar: 20 µm.

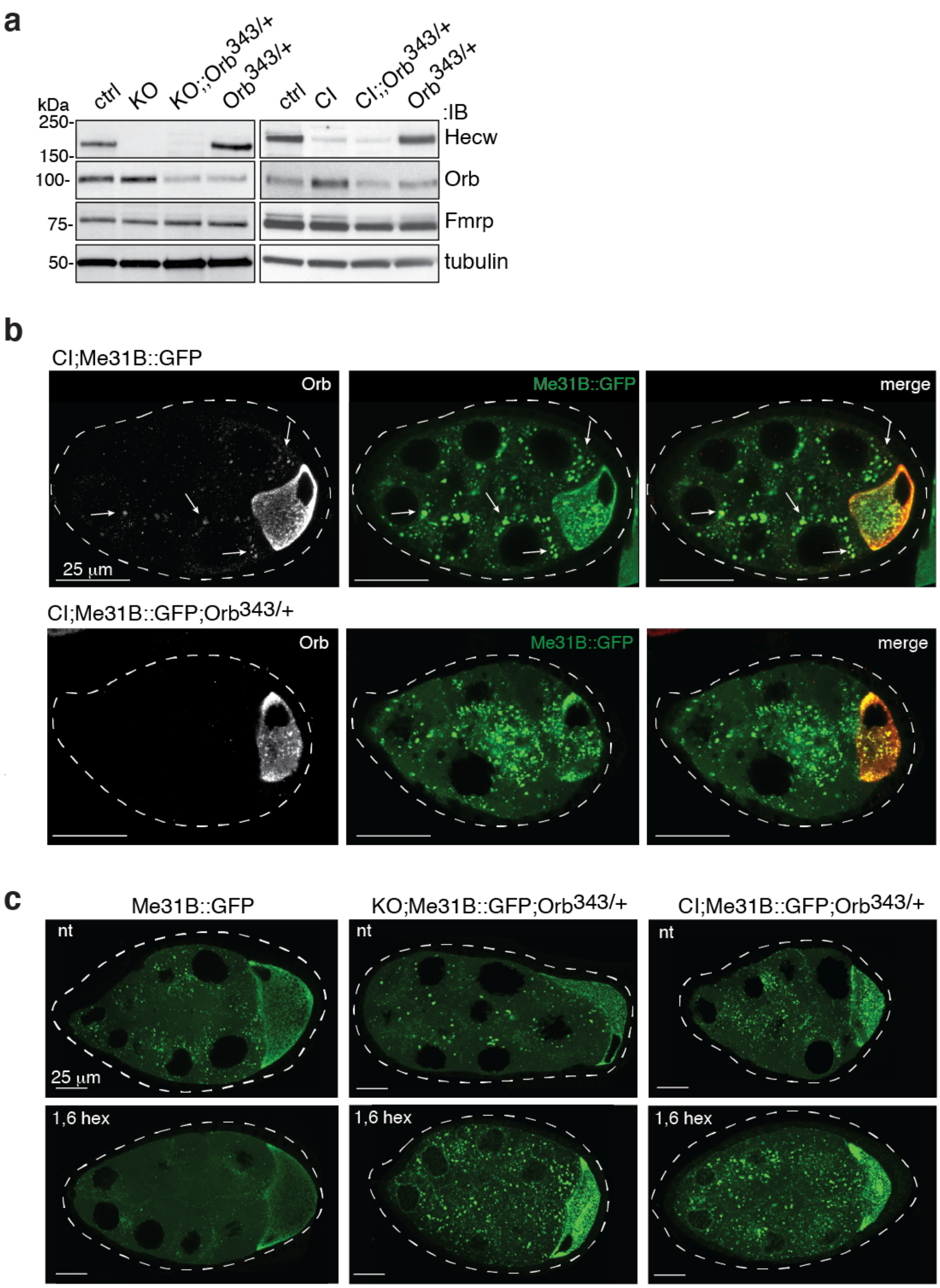

Supplementary Figure 8: Hecw^{CI} and Hecw^{KO} in the Orb heterozygous background.

(**a**) IB analysis of adult ovaries of the indicated genotypes. In the Orb heterozygous background, defective egg chambers phenotype scored in Hecw^{KO} and Hecw^{CI} homozygous flies is largely rescued (see Supplementary Table 1). (**b**) IF analysis of the indicated genotypes. Green,

Me31B::GFP which marks the RNPs; red, Orb which marks the oocyte (in white in the first panel). (n=2). Scale bar: 25 μm. In the Orb heterozygous background, Orb protein is no longer visible in the aberrant RNPs granules of the nurse cells. (**c**) IF analysis of egg chambers of 3-day-old flies as indicated, not treated (nt) or treated with 1,6 hexanediol (1,6 hex). (n=2). Scale bar: 25 μm. In the Orb heterozygous background, RNPs remained morphologically altered and solid-like even after the 1,6-hexanediol treatment.

Supplementary Tables

Genotype	flies age	NC, RC	Oo	Defects	Defective egg ch./tot (%)	
yw	3 day-old	15	1	/	0/310 (0%)	
$Hecw^{CI}$	3 day-old	7	1	mitosis	19/338 (6%)	$Hecw^{CI}$ 3-day-old tot: 21%
		7< n <15	1	mitosis, synchronization	28/338 (8%)	
		n> 15	1	mitosis, synchronization	6/338 (2%)	
		30	2	encapsulation	16/338 (5%)	
$Hecw^{KO}$	3 day-old	n<7	1	mitosis, synchronization	8/176 (5%)	$Hecw^{KO}$ 3-day-old tot: 19%
		7< n <15	1	mitosis, synchronization	4/176 (2,2%)	
		n> 15	1	mitosis, synchronization	5/176 (3%)	
		30	2	encapsulation	17/176 (9,7%)	
$Hecw^{KO::DC504}$	3 day-old	15	1	/	0/190 (0%)	
$Hecw^{CI};;Orb^{343}$	3 day-old	n<7	1	mitosis, synchronization	3/190 (1,6%)	$Hecw^{CI}Orb^{343}$ 3-day-old tot: 8%
		7< n <15	1	mitosis, synchronization	4/190 (2,1%)	
		n> 15	1	mitosis, synchronization	0/190 (0%)	
		30	2	encapsulation	8/190 (4,2%)	
$Hecw^{KO};;Orb^{343}$	3 day-old	n<7	1	mitosis, synchronization	2/260 (0,8%)	$Hecw^{KO}Orb^{343}$ 3-day-old tot: 3%
		7< n <15	1	mitosis, synchronization	0/260 (0%)	
		n> 15	1	mitosis, synchronization	0/260 (0%)	
		30	2	encapsulation	5/260 (2%)	
yw	30 day-old	15	1	/	0/150 (0%)	
$Hecw^{CI}$	30 day-old	7	1	mitosis	3/160 (2%)	$Hecw^{CI}$ 30-day-old tot: 39%
		7< n <15	1	mitosis, synchronization	11/160 (7%)	
		n> 15	1	mitosis, synchronization	10/160 (6%)	
		30	2	encapsulation	38/160 (24%)	
$Hecw^{KO}$	30 day-old	n<7	1	mitosis, synchronization	2/100 (2%)	$Hecw^{KO}$ 30-day-old tot: 37%
		7< n <15	1	mitosis, synchronization	6/100 (6%)	
		n> 15	1	mitosis, synchronization	7/100 (7%)	
		30	2	encapsulation	22/100 (22%)	
$Hecw^{KO::DC504}$	30 day-old	15	1	/	0/220 (0%)	
fmr^{D113}	3 day-old	n<7	1	mitosis, synchronization	9/178 (5%)	$fmr1^{\Delta 113}$ 3-day-old tot: 16%
		7< n <15	1	mitosis, synchronization	2/178 (1%)	
		n> 15	1	mitosis, synchronization	4/178 (2%)	
		30	2	encapsulation	14/178 (8%)	
tj>RFP Hecw	3 day-old	30	2	encapsulation	6/200 (3,5%)	
tj>RNAi Hecw	3 day-old	15	1	/	0/140 (0%)	
nos>RFP Hecw	3 day-old	n<>15	1	mitosis, synchronization	12/230 (5%)	nos> Hecw tot: 11%
		30	2	encapsulation	14/230 (6%)	
nos>RNAi Hecw	3 day-old	n<>15	1	mitosis, synchronization	10/210 (5%)	nos>RNAi Hecw tot: 13%
		30	2	encapsulation	16/210 (8%)	

Supplementary Table 1 Classification of oogenesis defects.

Complete classification of the defects observed in the indicated genotypes at the indicated time points. Numbers of nurse cells (NC), ring canals (RC), and oocytes (Oo) are specified in the third and fourth column, respectively.

gene name	human orth	function	WW inter motif
Hrb27C/Hrp48	*DAZAP1*	Heterogeneous nuclear ribonucleoprotein at 27C	PY
Fmr1	*FXR1*	Fragile X mental retardation syndrome-related protein	PR
bel	*DDX3X*	ATP-dependent RNA helicase bel	PR (x3)
larp	*LARP1B*	La-related protein 1, mRNA binding repressor	PR(x5), PY
Ef1alpha	*EEF1A2*	Elongation factor 1-alpha	PY
EF2	*EEF2*	Elongation factor 2	PR
CG10077	*DDX17*	ATP-dependent RNA helicase DEAD-box	PR (x2), PY
RpII215	*POLR2A*	DNA-directed RNA polymerase II subunit RPB1	PR(x5), PY
nito	*RBM15B*	spenito, mRNA binding protein, splicing	PR(x5), PY (x4)
lig	*UBAP2*	linger, mRNA binding protein	PR, PY
glo	*HNRNPH1/2*	glorund, mRNA binding protein	PY(x2)
nonA	*SFPQ*	No-on-transient A, putative RNA binding protein	PR (x3), PY
CG7878	*DDX43*	DEAD box RNA helicase	PR
unkempt	*UNK*	mRNA binding, ubiquitin	PPxY, PY(x2), PR(x3)
Hsc70-3	*HSPA5*	Heat shock 70-kDa protein cognate 3	PR (x2)
Hsp60	*HSPD1*	60 kDa heat shock protein, mitochondria	PY
Hsc70-4-RA	*HSPA8*	Heat shock protein cognate 4	/
CG7033	*CCT2*	Heat Shock Protein 60 chaperonins	PR
betetub56D	*TUBB4B*	Tubulin beta-1 chain	PR(x4), PY
betetub60D	*TUBB6*	Tubulin beta-3 chain	PR (x3), PY
Act5C	*ACTB*	Actin 5C	PR (x2)
zip	*MYH10*	myo II	PR, PY
Eb1	*MAPRE1*	Eb1, myosin and microtubule binding	PR
CG5787	-	microtubule associated complex	PR (x2), PY (x2)
RhoGAP15B	*ARAP3*	RhoGAP15B, isoform B	PR(x4), PY, PPXY (x2)
14-3-3zeta	*YWHAZ*	E3 adaptor, signaling	/
Ack	*TNK2*	Activated Cdc42 kinase	PR, PY
Rack1	*RACK1*	Receptor of activated protein kinase C 1	/
jub	*LIMD1*	Ajuba LIM protein	PR (x2), PY
l(1)G0193	-	Lethal (1) G0193	PR (x2)
CG3800	*CNBP*	zinc ion binding; nucleic acid binding	/
Vhc55	*ATP6V1B2*	Vacuolar H[+]-ATPase 55kD B subunit, isoform C	PR (x3)
Aldh	*ALDH2*	Aldehyde dehydrogenase	PY
blw	*ATP5F1A*	ATP synthase subunit alpha, mitochondrial OS	PY
ATPsyn-beta	*ATP5F1B*	ATP synthase subunit beta	PY
RpS3	*RPS3*	40S ribosomal protein S3	/
sta	*RPSA*	40S ribosomal protein SA	PR

Supplementary Table 2 Hecw interactor candidates.

List of Hecw interactors identified by mass spectrometry analysis. Red, proteins involved in mRNA binding and processing; Grey, cytoskeleton and motor proteins. Notably, 75% of the candidates (in bold) overlap with the recently identified interactome of the post-translational repressor Smaug in fly embryo[1].

Drosophila strains	Figure	Supplementary Figure
Dp(1;3)DC504	3d	
elav-GAL4	8e	3d-f
elav-GAL4;;UAS-RFPHecw	8a,e	3d-f
elav-GAL4;UAS-Dicer2/UAS-Fmr1RNAi;UAS-RFPHecw	8e	
fmr1$^{\Delta 113M}$	7b	
Hecw^{CI}	2a,c-e; 3a,b; 7b,f,h,i	2a-c; 3a-c; 4a,b; 7c; 8a
Hecw^{CI}*;;Orb*$^{343/+}$		8a
Hecw^{CI} *;;fmr* $^{\Delta 113}$		7b
Hecw^{KO}	2b-f; 3a,b,d; 4a; 7e,f,i; 8a	2d,e; 4a,b; 7c; 8a
Hecw^{KO}*;;Orb*$^{343/+}$		8a
Hecw$^{KO;;DC504}$	2d,e,g; 3d	
Hecw^{CI} *;Me31B::GFP*	4 b,c,d,e; 8c,d	8b
Hecw^{KO} *;Me31B::GFP*	4b-e; 5 a-d; 6a-d; 7a; 8b-d	5a,c-i; 6a-d; 7a,f
Me31B::GFP	4b-e; 5 a-d; 6a-d; 7a; 8b-d	5a-i; 6a-d; 7a,f; 8c
nanosGAL4-VP16	3c,e; 7g;8e	7d,e
nanosGAL4-VP16;;UAS-HecwRNAi	3c	4a
+;UAS-Fmr1RNAi/Dicer2;nanosGAL4/UAS-Hecw	8e	
nanosGAL4-VP16;;UAS-RFPHecw	3e; 7g;8e	7d,e
traffic jam-GAL4		1c; 4c,d
traffic jam-GAL4; UAS-HecwRNAi		4c
traffic jam-GAL4; UAS-RFPHecw		1c; 4d
y^{1}*w*^{1}	1b-e; 2a-g; 3a,b,d; 4a; 7a-c,e,f,h,i; 8a	1f,g; 2b-e; 3a-c; 4a,b; 7a-c; 8a

Supplementary Table 3 Genotypes of strains used in figures.

Primer name	Primer sequence
Hecw^{CI}	5’-CTTCGTGCCCACACATGCTTCAAT-3’,<br>5’-AAACAATGAAGCATGTGTGGGCAC-3’
Hecw^{KO}	5’-CTTCGCCTTCTACGAGGCGCGCAA-3’,<br>5’-AAACTTGCGCGCCTCGTAGAAGGC-3’
Hecw^{CI}_F	5’-CCGAGAGTTGGAGCTGGTTA-3’
Hecw^{CI}R	5’-AAACTAGTGGGATGCCATGC-3’
Hecw^{KO}_F	5’-ATGGAGCCACCAGCT-3’
Hecw^{KO}_R	5’-AGCTGGTGGCTCCAT-3’
CG42797 BglII_F	5’-GTCCGGACTCagatctATGGAGCCACCAGCTGCA-3’
CG42797 XhoI_R	5’-TAGAGGTACCctcgagCTACTCAATGCCGAACGTGTTG-3’
CG42797 EcoRI_F	5’-CCGgaattcATGGAGCCACCAGCT-3’
C1394W_F	5’-CCCGTGCCCACACATGGTTCAATCGGCTGGATTTG-3’
C1394W_R	5’- CAAATCCAGCCGATTGAACCATGTGTGGGCACGGG-3’
CG42797 EcoRI WWI_F	5’-ccgGAATTCCCACCATTGCCGCCTG-3’
CG42797 XhoI WWII_R	5’- ccgCTCGAGTCAACGAGGATCCATGAA-3’
CG42797 XhoI_Nterm_R	5’- CCGctcgagTCATTCGCTGGGCTGC-3’
Fmrp BamH_F	5’-CGCggatccATGGAAGATCTCCTC-3’
Fmrp EcoR1_R	5’-CCGgaattcTTAGGACGTGCCATT-3’
387_dFMRP_F	5’-GCggatccCAGGAATCCTCGATGGGC-3’
dFMRP_NcoI_R	5’- CATCccatggCGGACGTGCCATTGACC-3’
NcoI_Ub_F	5’ -CATGccatggGAACGGGCAGTACGGGCAGTATGCAGATCTTCGT CAAGACG- 3’
Ub_SmaI_R	5’- TCCcccgggGGATCAACCACCTCTTAGTCTTAAGAC-3’

Supplementary Table 4 List of primers used in this study.

Supplementary Reference:

1 Cao, W. X. *et al.* Precise Temporal Regulation of Post-transcriptional Repressors Is Required for an Orderly Drosophila Maternal-to-Zygotic Transition. *Cell Rep* **31**, 107783, doi:10.1016/j.celrep.2020.107783 (2020).
