# Supplementary material for: Hecw controls oogenesis and neuronal homeostasis by promoting the liquid state of ribonucleoprotein particles
Source: Nat Commun. 2021 Sep 16;12:5488. doi: 10.1038/s41467-021-25809-8 (PMC8446043; doi:10.1038/s41467-021-25809-8)
Supplement: Supplementary file 3 — Reporting Summary [file 41467_2021_25809_MOESM3_ESM.pdf]

nature portfolio

Corresponding author(s): Thomas Vaccari, Elena Maspero, SImona Polo

Last updated by author(s): 2021/07/07

Reporting Summary

Nature Portfolio wishes to improve the reproducibility of the work that we publish. This form provides structure for consistency and transparency in reporting. For further information on Nature Portfolio policies, see our Editorial Policies and the Editorial Policy Checklist.

Statistics

For all statistical analyses, confirm that the following items are present in the figure legend, table legend, main text, or Methods section.

n/a	Confirmed	
☐	☒	The exact sample size (*n*) for each experimental group/condition, given as a discrete number and unit of measurement
☐	☒	A statement on whether measurements were taken from distinct samples or whether the same sample was measured repeatedly
☐	☒	The statistical test(s) used AND whether they are one- or two-sided<br>*Only common tests should be described solely by name; describe more complex techniques in the Methods section.*
☒	☐	A description of all covariates tested
☐	☒	A description of any assumptions or corrections, such as tests of normality and adjustment for multiple comparisons
☐	☒	A full description of the statistical parameters including central tendency (e.g. means) or other basic estimates (e.g. regression coefficient) AND variation (e.g. standard deviation) or associated estimates of uncertainty (e.g. confidence intervals)
☒	☐	For null hypothesis testing, the test statistic (e.g. *F*, *t*, *r*) with confidence intervals, effect sizes, degrees of freedom and *P* value noted<br>*Give P values as exact values whenever suitable.*
☒	☐	For Bayesian analysis, information on the choice of priors and Markov chain Monte Carlo settings
☒	☐	For hierarchical and complex designs, identification of the appropriate level for tests and full reporting of outcomes
☒	☐	Estimates of effect sizes (e.g. Cohen's *d*, Pearson's *r*), indicating how they were calculated

Our web collection on statistics for biologists contains articles on many of the points above.

Software and code

Policy information about availability of computer code

Data collection	Fixed images were acquired with a Nikon ECLIPSE C1si (Tunel assay) or a Leica TCS Sp2 upright confocal microscope.<br>Live cell imaging experiments were performed on the UltraVIEW VoX spinning-disk confocal system (PerkinElmer), equipped with an EclipseTi inverted microscope (Nikon), a Hamamatsu CCD camera (C9100-50) and driven by Volocity software (Improvision; Perkin Elmer).<br>For FRAP analysis, time series were recorded using a Confocal Spinning Disk microscope (Olympus) equipped with IX83 inverted microscope provided with an IXON 897 Ultra camera (Andor) and a IX3 FRAP module.<br>FLIP experiments were performed on the UltraVIEW VoX spinning-disk confocal system (PerkinElmer) equipped with an EclipseTi inverted microscope (Nikon), provided with an integrated FRAP PhotoKinesis unit (PerkinElmer) and a Hamamatsu CCD camera (C9100-50) and driven by Volocity software (Improvision; Perkin Elmer).<br>Mass spectrometry analysis was performed with the Q Exactive HF-X Hybrid Quadrupole-Orbitrap (Thermo Fisher).
Data analysis	Images were analysed with FIJI Software (https://imagej.net/Fiji version 2.1.0/1.53c), Volocity software (Improvision; Perkin Elmer, version 6.3). Particles were tracked with the Manual tracking plugin of Image J and analysed with the Chemotaxis plugin (https://ibidi.com/chemotaxis-analysis/171-chemotaxis-and-migration-tool.html). The remaining homemade developed analysis (DDM, Fiji plugins) were extensively described in the methods section of the paper.<br>Statistical analyses were performed with Prism (GraphPad software version 9.1.).<br>Proteomic raw data were processed using Proteome Discoverer (version 1.4.0.288, Thermo Fischer Scientific), Scaffold (version Scaffold_4.3.4, Proteome Software Inc., Portland, OR) and Peptide Prophet algorithm.

For manuscripts utilizing custom algorithms or software that are central to the research but not yet described in published literature, software must be made available to editors and reviewers. We strongly encourage code deposition in a community repository (e.g. GitHub). See the Nature Portfolio guidelines for submitting code & software for further information.

Data

Policy information about availability of data

All manuscripts must include a data availability statement. This statement should provide the following information, where applicable:

- Accession codes, unique identifiers, or web links for publicly available datasets
- A description of any restrictions on data availability
- For clinical datasets or third party data, please ensure that the statement adheres to our policy

The mass spectrometry raw datasets generated in this study have been deposited in PRIDE database and can be accessed through ProteomeXchange with the following Project Name: Pulldown CG42797 vs S2 cell lysate and Project accession: "PXD025809". Full list of the specific interactors is provided in Supplementary Table 2. All other relevant data are available from the authors. Source data are provided with the paper.

Field-specific reporting

Please select the one below that is the best fit for your research. If you are not sure, read the appropriate sections before making your selection.

☒ Life sciences ☐ Behavioural & social sciences ☐ Ecological, evolutionary & environmental sciences

For a reference copy of the document with all sections, see nature.com/documents/nr-reporting-summary-flat.pdf

Life sciences study design

All studies must disclose on these points even when the disclosure is negative.

Sample size	No statistical methods were used to determine sample size. Typical sample size in the field is 6-30 animals or organ explants, expect for lifespan assay that were conducted with at least 100 animals/genotypes. All animals analyzed were from low density cultures (approximately 50 animals per vial) and sample size was determined based on our initial observations of phenotypic penetrance. Sample size refers to number of animals, brains, ovaries or egg chambers and are reported as N for each experiment and described in their relative figure legend.
Data exclusions	No data points were excluded in this work. All data points were represented in the figures and used in statistical analyses.
Replication	Data was successfully replicated in independent experiments (at least three independent experiments with three technical replicates when variation among genotypes were visible and at least two with three technical replicates when no changes were evident) and can be reproduced by independent investigators.
Randomization	Flies were sampled randomly for each experiment and were grouped according to genotype
Blinding	Researchers were blinded to group allocation during immunofluorescence experiments. For other experiments, we relied upon replication and confirmations of results by at least 2 independent techniques or investigators when possible. Randomization or blind analysis is impossible for Wester blot analysis. Western blots were run with all conditions in parallel in the same gel.

Reporting for specific materials, systems and methods

We require information from authors about some types of materials, experimental systems and methods used in many studies. Here, indicate whether each material, system or method listed is relevant to your study. If you are not sure if a list item applies to your research, read the appropriate section before selecting a response.

Materials & experimental systems

n/a	Involved in the study	
☐	☒	Antibodies
☐	☒	Eukaryotic cell lines
☒	☐	Palaeontology and archaeology
☐	☒	Animals and other organisms
☒	☐	Human research participants
☒	☐	Clinical data
☒	☐	Dual use research of concern

Methods

n/a	Involved in the study	
☒	☐	ChIP-seq
☒	☐	Flow cytometry
☒	☐	MRI-based neuroimaging

Antibodies

Antibodies used	The antibodies used in this study are listed here: rat anti-elav antibody (IF 1:50; DSHB 7E8A10), rabbit anti-Hecw (IB 1:250, IF 1:200 , IP 4µg/mg; generated in house), Llama GFP-TRAP_A (Chromotek gta-20), rabbit anti-GFP (IB 1:5000; Sigma G1544), mouse anti-Fmrp (IB 1:300, IF 1:300, IP 3µg/mg; DSHB 5A11-s), mouse anti-Profilin (IB 1:200, DSHB chi-1J); mouse anti-Grk (IB 1:400, IF

March 2021

1:400; DSHB 1D12-s), rabbit anti-K63 ubiquitin (IF 1:250; Millipore clone Apu-3 05-1308), rabbit anti-K48 ubiquitin (IF 1:250; Millipore clone Apu-2 05-1307), mouse anti-Orb (IB 1:250, IF 1:250; DSHB 4H8-s), mouse anti-Orb (IB 1:250, IF 1:250, IP 1µg/mg; DSHB 6H4-s), rabbit anti-Oskar (IF 1:2000; Ephrussi lab), mouse anti-ubiquitin (IF 1:300, IP 20µg/mg; Enzo Life Science clone FK2 BML-PW8810), mouse anti-ubiquitin (IB 1:5; generated in house, clone ZTA10), rat anti-alpha tubulin (IF 1:100; Bio-Rad MCA78G), mouse anti-tubulin (IB 1:1000; Sigma T5168), HRP-conjugated goat anti-mouse IgG (IB 1:10000; Bio-Rad 1721011), HRP-conjugated goat IgG (IB 1:10000; Bio-Rad 1706515), Alexa647-conjugated donkey anti-rabbit (IF 1:400; Thermo Fisher A31571), Alexa488-conjugated donkey anti-rabbit (IF 1:400; Thermo Fisher A21206), Alexa647-conjugated goat anti-rat (IF 1:400; Thermo Fisher A21247), Alexa488-conjugated donkey anti-mouse (IF 1:400; Thermo Fisher A21202), Cy3-conjugated donkey (IF 1:400; Jackson Lab 715-165-150).

Validation

The anti-Hecw was produced by Eurogentech S.A in rabbit using the N-terminal fragment of Hecw (aa 1-130) fused to GST protein. The resulting polyclonal antibody was affinity purified and validated on S2 cells depleted of Hecw as described in Supplementary Figure 1.

1) anti-elav antibody (7E8A10 from DHSB). Description and validation information at https://antibodyregistry.org/search.php?q=AB_528218
2) GFP-TRAP Chromotek gta-20. Description: https://www.chromotek.com/products/detail/product-detail/gfp-trap-agarose/ reference: PMID: 24906338
3) rabbit anti-GFP (Sigma G1544). Description: https://www.sigmaaldrich.com/IT/it/product/sigma/g1544 reference: PMID: 17287248
4) mouse anti-Fmrp (5A11-s from DSHB). Description and validation information at https://antibodyregistry.org/search.php?q=AB_10805421
5) mouse anti-Profilin (chi-1J from DSHB). Description and validation information at https://antibodyregistry.org/search.php?q=AB_528439
6) mouse anti-Grk (1D12 form DSHB). Description and validation information at https://antibodyregistry.org/search.php?q=AB_528273
7) rabbit anti-K63 ubiquitin (Millipore clone Apu-3 05-1308). Description and references: https://www.merckmillipore.com/IT/it/product/Anti-Ubiquitin-Antibody-Lys63-Specific-clone-Apu3-rabbit-monoclonal,MM_NF-05-1308
8) mouse anti-Orb (DSHB 4H8). Description and validation information at https://antibodyregistry.org/search.php?q=AB_528418
9) mouse anti-Orb (DSHB 6H4). Description and validation information at https://antibodyregistry.org/search.php?q=AB_528419
10) rabbit anti-Oskar (generated by the Ephrussi lab). Description and validation in PMID: 26190108
11) mouse anti-ubiquitin (Enzo Life Science clone FK2 BML-PW8810). Description and validation information at https://www.citeab.com/antibodies/307994-bml-pw8810-ubiquitin-fk2
12) mouse anti-ubiquitin (generated in house, clone ZTA10). Description and validation in PMID26971995
13) rat anti-alpha tubulin (Bio-Rad MCA78G). Description and validation information at https://www.bio-rad-antibodies.com/monoclonal/yeast-tubulin-alpha-antibody-yol1-34-mca78.html
14) mouse anti-tubulin (Sigma T5168). Description and validation information at https://www.citeab.com/antibodies/2304940-t5168-monoclonal-anti-alpha-tubulin-antibody-produce

The remaining secondary antibodies were validated by the manufacturers, as shown in their respective datasheets.

Eukaryotic cell lines

Policy information about cell lines

Cell line source(s)	Drosophila S2 cells obtained from Invitrogen
Authentication	The cell line was not authenticated as was directly purchased from the company and was grown in isolated room.
Mycoplasma contamination	Cells are routinely tested for mycoplasma and were always mycoplasma free.
Commonly misidentified lines (See ICLAC register)	Not applicable

Animals and other organisms

Policy information about studies involving animals; ARRIVE guidelines recommended for reporting animal research

Laboratory animals	A full list of all the genotypes of the Drosophila strains used in the study is listed in Supplementary Table 3. Animals were not separated by sex for lifespan assay and climbing assay, while female flies were used to analyse the ovaries.
Wild animals	No wild animals were used in the study.
Field-collected samples	No field-collected samples were used in the study.
Ethics oversight	This study did not require an ethical approval.

Note that full information on the approval of the study protocol must also be provided in the manuscript.
