# Supplementary material for: Hecw controls oogenesis and neuronal homeostasis by promoting the liquid state of ribonucleoprotein particles
Source: Nat Commun. 2021 Sep 16;12:5488. doi: 10.1038/s41467-021-25809-8 (PMC8446043; doi:10.1038/s41467-021-25809-8)
Supplement: Supplementary file 4 — Description of Additional Supplementary Files [file 41467_2021_25809_MOESM4_ESM.pdf]

Description of Additional Supplementary Files

Supplementary Movie 1 Live imaging of Me31B::GFP in wild type egg chambers.

Supplementary Movie 2 Live imaging of Me31B::GFP in *HecwKO;Me31B::GFP* egg chambers.

Supplementary Movie 3 Fusion of Me31B::GFP particles in wild type egg chambers.

Supplementary Movie 4-5 Representative of FLIP analysis performed on Me31B::GFP in wild-type egg chambers.

Supplementary Movie 6-7 Representative of FLIP analysis performed on Me31B::GFP in *HecwKO;Me31B::GFP* egg chambers.
